# Advancements in Detection Methods for *Salmonella* in Food: A Comprehensive Review

**DOI:** 10.3390/pathogens13121075

**Published:** 2024-12-07

**Authors:** Aayushi Patel, Andrew Wolfram, Taseen S. Desin

**Affiliations:** 1 Trinity School of Medicine, Trinity Medical Sciences University, Roswell, GA 30075, USA; aayushi.patel.22@tmsu.edu.vc (A.P.); andrew.wolfram.22@tmsu.edu.vc (A.W.); 2 Department of Medical Education, College of Medicine, University of Central Florida, Orlando, FL 32827, USA

**Keywords:** food safety, pathogen detection, non-typhoidal *Salmonella*, aptasensors, surface plasmon resonance, Raman Spectroscopy

## Abstract

Non-typhoidal *Salmonella* species are one of the leading causes of gastrointestinal disease in North America, leading to a significant burden on the healthcare system resulting in a huge economic impact. Consequently, early detection of *Salmonella* species in the food supply, in accordance with food safety regulations, is crucial for protecting public health, preventing outbreaks, and avoiding serious economic losses. A variety of techniques have been employed to detect the presence of this pathogen in the food supply, including culture-based, immunological, and molecular methods. The present review summarizes these methods and highlights recent updates on promising emerging technologies, including aptasensors, Surface Plasmon Resonance (SPR), and Surface Enhanced Raman Spectroscopy (SERS).

## 1. Introduction

Non-typhoidal *Salmonella* (NTS) is a ubiquitous Gram-negative bacterial pathogen and among the leading causes of food and water-borne illnesses worldwide [1]. It causes an estimated 1.35 million infections in the United States annually, second only to Norovirus as the most prevalent causative agent for foodborne diseases [1]. NTS is the leading cause of foodborne infection-related hospitalizations and deaths in the United States, responsible for 26,500 hospitalizations and approximately 420 deaths each year [1]. The European Food Safety Authority (EFSA) has estimated that the overall economic burden of human salmonellosis could be as high as EUR 3 billion per year [2]. A member of the Enterobacteriaceae family, the bacterial genus *Salmonella* consists of two species, *Salmonella enterica* and *Salmonella bongori*, of which *Salmonella enterica* is further divided into six subspecies, with *Salmonella enterica* subspecies *enterica* consisting of at least 1531 serovars [3]. NTS harbors in the intestines and feces of animals and is primarily acquired through the consumption of contaminated foods, such as undercooked meat, raw eggs, dairy products, or fresh produce [4,5,6,7]. Of particular importance are poultry products, as chickens are a significant reservoir for NTS [8]. The most commonly implicated serovars in the case of human salmonellosis, according to the CDC, are *S. enterica* subspecies *enterica* serovar Typhimurium (*S*. Typhimurium), *S.* Enteritidis, and *S.* Newport [9,10].

Salmonellosis typically manifests as gastroenteritis characterized by diarrhea, abdominal cramps, nausea, vomiting, and fever [11,12]. Immunocompromised individuals, children under the age of five, and the elderly are particularly susceptible to infection [13,14]. The infection has an incubation period ranging from 6 to 96 h [15,16]. The disease is usually self-limited, resolving within 10 days in most healthy adults [11,12,13,14].

Upon ingestion, *Salmonella* survives its passage through the gastrointestinal tract, invades the M-cells of the intestinal epithelium, and replicates intracellularly, aided by Type 3 Secretion Systems (T3SS) encoded on *Salmonella* Pathogenicity Islands (SPI) [17,18,19,20]. In approximately 5% of *Salmonella* infections, the bacteria can spread from the intestine into the bloodstream, resulting in bacteremia. Additionally, in about 1.8% of cases of bacteremia, extraintestinal complications can manifest largely due to the persistence of *Salmonella* inside tissue macrophages [21]. According to the European Commission, the presence of *Salmonella* at 1 colony forming unit per milliliter (CFU/mL) in a portion of 25 g of ready-to-eat food is sufficient to cause disease in humans [1,9]. Early detection of *Salmonella* species in contaminated food is necessary to prevent human infections, allowing for at least 27 food recalls in 2024 by the U.S. Food and Drug Administration (FDA), as this pathogen is a public health concern and poses a significant threat to the safety of our food supply [1,22]. Additionally, timely detection of this pathogen results in the prevention of significant economic losses due to product recalls, medical expenses, and reduced consumer confidence in food safety [23]. Accurate detection of the *Salmonella* genus in food products also ensures compliance with various regulations and standards in different countries, which assists in maintaining the integrity of the food industry and avoids legal repercussions. In the USA, the validation of a *Salmonella* detection method involves a rigorous process that complies with guidelines set by regulatory agencies like the FDA, the U.S. Department of Agriculture (USDA), and sometimes the Environmental Protection Agency (EPA) [24]. Additionally, the requirements must also align with international standards, such as the International Organization for Standardization (ISO), particularly ISO 16140, which provides a framework for validating microbiological methods for food testing [25]. Similarly, in the European Union (EU), the validation of a *Salmonella* detection method must comply with standards set by the European Food Safety Authority (EFSA), Regulation (EC) No 2017/625 and ISO 16140 [26,27]. Moreover, with the increase in global food trade, it is essential to detect *Salmonella* in order to safeguard global food security and minimize the risk of spreading *Salmonella* across borders. A multitude of techniques are currently used to detect the bacterial pathogen *Salmonella*, and this review attempts to highlight new, promising technologies that can be used for this purpose.

## 2. Traditional Culture-Based Methods

Culture-based detection remains the ‘gold standard’ for microbiological analysis due to its high success rate and cost-effectiveness [1,9,28,29]. It can detect viable *Salmonella* in a 25 g sample with a detection limit of 1 CFU [29,30]. Since culture has been used to detect bacteria for so long, it is often required by law to detect foodborne pathogens via culture despite more modern detection methods being available [9]. Culture-based methods are sensitive, inexpensive, and can indicate the number and type of viable microorganisms in food samples. However, culture-based detection has several limitations, including slow growth rates (24–72 h) and labor-intensive steps [31].

The current procedure outlined by the International Organization for Standardization, ISO 6579-1:2017, involves non-selective enrichment in Buffered Peptone Water for approximately 18 h, followed by selective enrichment in Rappaport Vassiliadis broth or Muller-Kauffmann Tetrathionate Novobiocin broth for approximately 24 h [30]. Isolation of *Salmonella* colonies is done by plating on Xylose Lysine Deoxycholate (XLD) Agar and a second selective isolation agar complementary to XLD Agar as described in Annex E of ISO 6579-1:2017 [30]. This step requires another 24 h. Finally, biochemical identification and serological testing are performed, which can take an additional three days. However, with the use of rapid biochemical assay kits, multiple assays can be performed simultaneously, and results can be acquired within a day of inoculation [32]. More recently, an automated Biolog microbial identification system was developed, which uses redox indicators and imaging technology to identify multiple bacterial strains at the same time [33]. Additionally, serotyping is done for further typing of the *Salmonella* strains to the serovar level according to ISO/TR 6579-3:2014 [34]. It involves the use of standard agglutination methods using polyclonal antibodies against O antigens, flagellar H antigens, and capsular Vi antigens to identify the specific serogroup of *Salmonella* species. However, this poses a challenge for detection in fermented and heat-treated foods, as the surface antigens are often modified or missing, leading to false negatives [35]. Overall, agglutination tests are laborious, and often even acquiring the required antisera is time-consuming [32,35,36].

## 3. Immunological Techniques

Various immunological methods have been developed for the detection of *Salmonella*, such as enzyme-linked immunosorbent assays (ELISA) and lateral flow immunoassays, which utilize antigen–antibody interactions to offer rapid and specific identification suitable for both clinical and field applications [36]. Immunoassays use anti-*Salmonella* polyclonal antibodies fixed on a solid matrix that are bound to a chromogenic substrate [37]. Upon binding a specific *Salmonella* antigen to its corresponding antibody, an enzymatic reaction is triggered, producing a color change. ELISAs are one of the most commonly used immunoassays which have the potential of producing false positives due to the cross-reactivity of polyclonal antibodies. Additionally, ELISA’s poor sensitivity (10^6^ CFU/mL) can be enhanced by using monoclonal antibodies, which are more specific, but costly and complex to produce [38]. ELISA also benefits from color changes visible to the naked eye, facilitated by tetramethylbenzidine (TMB) and p-nitrophenyl phosphate (pNPP). Sandwich ELISA and Duplex PCR-ELISA have higher sensitivity (1 CFU/mL) and specificity and are useful for the detection of *Salmonella* as well as other foodborne pathogens [39]. Novel methods, such as bispecific antibodies and DNAzymes (man-made DNA molecules), have also been developed for more efficient pathogen detection. Monoclonal IgY antibodies from chicken egg yolk are advantageous due to ease of purification and high yield. Other advanced methods include gold-labeled immunosorbent assays (GLISA), which offer lower detection limits and faster results through enrichment steps, and label-free immunoassays that enable simultaneous enrichment and detection [40].

Lateral Flow immunochromatographic devices allow for a sample to flow, via capillary action, through a solid substrate to a bioreceptor conjugated with a colorimetric label, which has been shown to be effective as a proof of principle for the detection of the lipopolysaccharides (LPS) of *Salmonella* without the need for pre-enrichment [41]. They are rapid, relatively inexpensive, and easy to use. The analyte is placed on the sample pad and filtered through. It can then bind nanoparticles in the Lateral Flow Assay (LFA) strip and flow through the membrane, reaching the anti-*Salmonella* antibodies. Upon binding of a *Salmonella* antigen, a color change is produced, forming the test line. Beyond the test line, there are additional bio-receptors used to validate the LFA device which detect nanoparticles regardless of bound analyte and form the control line. In 2021, using a proof-of-concept approach, Gao et al. developed LFA strips using aptamer-magnetic separation and nanoparticles for enhanced detection of *S*. Typhimurium in milk with a limit of detection of 4.1 × 10^2^ CFU/mL without a pre-enrichment step [42].

## 4. Molecular Methods

A variety of rapid molecular methods have been developed for the detection of *Salmonella*, including PCR-based assays, nucleic acid amplification (NAA) tests, and next-generation sequencing (NGS) [40]. Polymerase chain reaction (PCR) is used to detect and amplify specific DNA or RNA sequences of pathogens using designed primers. PCR is widely used due to its speed, high sensitivity, specificity, reliability, and potential to be automated compared to most culture-based and immunoassay detection methods [40].

In 2020, Chirambo et al. conducted a prospective cohort study on 50 asymptomatic Malawian children to determine a reliable molecular diagnostic test for *Salmonella* in stool specimens using *ttr* and *invA* as target genes [43]. Many variants of this approach have been used to successfully detect *Salmonella* in cheese, milk, beef, salad, fish, pork, chicken, and egg products, including nested PCR, touchdown PCR, multiplex PCR, reverse transcriptase PCR (RT-PCR), real-time or quantitative PCR (qPCR), droplet digital PCR (ddPCR), and viable PCR (vPCR) [44]. Additionally, real-time or quantitative PCR allows for the detection and quantification of viable target organisms, which is not the case in conventional PCR [42]. Furthermore, methods such as RT-PCR are promising, yet challenging, as mRNA may be rapidly degraded in food matrices [45]. *Salmonella* screening via real-time PCR is typically completed within 24–48 h of sample collection due to a lengthy enrichment phase to grow the pathogen in a proper medium [40]. Reverse transcriptase PCR converts mRNA into complementary DNA used as a template for exponential amplification. RT-PCR is a typical first choice for viral pathogen detection. Despite mRNA being a better indicator of bacterial viability, its short half-life leads to false negative results of RT-PCR in bacterial detection [45]. In 2021, Zendrini and colleagues found qPCR to have a limit of detection of 10 CFU/g of *Salmonella* in ground chicken meat after just 4 h of enrichment [46]. One major drawback of most PCR variants, including qPCR, is how they amplify DNA from both dead and viable bacteria. Viable PCR improves the detection of cell viability and reduces false negative results by using intercalating dyes such as ethidium monoazide (EMA) and propidium monoazide (PMA). These dyes penetrate the membranes of damaged or dead bacterial cells, binding irreversibly to their DNA [46]. This binding prevents the DNA from being amplified by PCR primers, ensuring that only DNA from live bacterial cells is amplified [47].

In recent years, other amplification techniques have been developed to improve detection speed, sensitivity, and affordability compared to PCR [48]. Loop-mediated isothermal amplification (LAMP) is an isothermal reaction that requires ⅔ pairs of primers and a DNA polymerase with high strand displacement to amplify the target region through the elongation via a hairpin structure with stem-loops at each end [49]. Despite the complexities of setup and optimization of LAMP compared to PCR, LAMP has several advantages, including eliminating a thermal cycler, higher and faster amplification (~1 billion copies LAMP vs. ~1 million copies in normal PCR in 60 min), less sensitivity to inhibitors like detergents, salts, and lipids, and amplification that can be observed at a glance [46]. In 2021, Zendrini et al. evaluated the effect of short enrichment periods of 0, 2, 4, and 6 h combined with real-time PCR or colorimetric LAMP to establish a one-day workflow to detect *Salmonella* and *Campylobacter* in poultry meat at different concentration levels. They reported that both LAMP and PCR detected *Salmonella* in chicken meat down to 10 CFU/g and 10^3^ CFU/g, respectively, without enriching their samples [46]. Moreover, several studies have reported that LAMP technology enabled the detection of *Salmonella* in chicken and pork that could be observed with the naked eye via turbidity anomaly or colorimetric changes during the amplification process, hinting at its on-site detection suitability [50,51,52].

Among the amplification techniques used for the detection of *Salmonella* species, isothermal amplification methods offer rapid detection without thermal cycles. However, issues of reagent stability and background signal interference still exist [51]. The advantages and disadvantages of many isothermal amplification methods, including Recombinase Polymerase Amplification (RPA), Recombinase Aided Amplification (RAA), Nucleic Acid Sequence-Based Amplification (NASBA), and Single Primer Isothermal Amplification (SPIA), are listed in Ndraha et al., 2023 [53]. Both RPA and RAA utilize recombinase enzymes to form primer complexes that scan DNA for homologous sequences and create D-loop structures. They are typically completed within 30 min and can operate at low temperatures (37–42 °C). The advantages of RPA and RAA are their rapid ability to detect various pathogens in food samples, often without the need for DNA enrichment [53]. Additionally, in 2021, Mu and colleagues found that RAA detected *Salmonella* Typhimurium in chicken meat at 10 CFU/mL without enrichment [54]. However, RPA and RAA can be labor-intensive, and the recombinase-primer complex can be expensive. Similarly, NASBA is sensitive and specific, without the need for denaturation steps, but requires complex equipment and poses challenges for those handling RNA. SPIA, likewise, is highly specific and sensitive but involves a complicated experimental procedure and utilizes expensive enzymes [53].

Rolling Circle Amplification (RCA) is another nucleic acid amplification (NAA) technique that utilizes a circular template to generate a lengthy single-strand product [55]. Studies have shown that RCA has high throughput detection, sensitivity, specificity, and application in pathogen detection. However, background interference and food matrices have proven to be a challenge during signal detection [55]. Researchers have reported that Saltatory Rolling Circle Amplification (SRCA) outperforms PCR in detecting pathogens in food samples [55]. SRCA has high specificity, sensitivity (4–40 CFU/g), a visual assessment of results, and is cheaper than LAMP, NASBA, and SPIA. However, the limited availability of SRCA in commercial kits, along with a complex primer selection process, are current drawbacks with this approach that require further refinement and research [53].

DNA microarrays form the basis of an important tool that can be used for the detection of *Salmonella* species in food samples. This involves the detection of the expression of thousands of genes simultaneously by extracting RNA from cells and converting it into cDNA [56]. The cDNA is then labeled with fluorescent dyes and hybridized to detect fluorescence, indicating the quantity of specific gene sequences to analyze gene expression. This technology is commercially available as PathogenDx [57] and has been validated by the Association of Analytical Chemists (AOAC) as AOAC-PTM #092001 for the detection of *Salmonella* in ground beef [58]. Recently, Delgado and colleagues used PathogenDx microarray to detect the *invA* gene of *Salmonella* in ground beef [56]. They established that their system could complete an enrichment step in 8 h, labeling, hybridization, and analysis within 12.5 h, and was able to correctly identify *Salmonella* in 93.33% and 100% of the samples when ground beef was inoculated with 1 and 5 CFU/g, respectively [56]. This study highlights the rapid and accurate detection of *Salmonella* using DNA microarray technology in ground beef. However, future studies on *Salmonella* detection using DNA microarrays with more rapid enrichment of other food media like poultry, dairy products, and fresh produce are needed.

Whole Genome Sequencing (WGS) is a process that involves sequencing the entire genome of an organism, which involves the use of technologies such as NGS, which have been effectively employed for *Salmonella* detection in food samples [59]. NGS technologies were launched in 2000 by Lynx Therapeutics to rapidly sequence large amounts of DNA or RNA [60]. Since its launch, numerous sequencing methods have been developed, including the 454 Roche method, sequencing by oligonucleotide ligation and detection (SOLiD), and the Illumina Solexa DNA sequencing system [60]. WGS is routinely used by the FDA for the detection of foodborne pathogens like *Salmonella* during routine surveillance and part of outbreak investigations [61]. Additionally, with the establishment of open access genomic reference databases such as GenomeTrackr, WGS can be effectively used for pathogen identification [62]. The aforementioned technologies provide reliable and accurate processing of many samples simultaneously, with predictable turn-around times for vast amounts of useful data in pathogen surveillance, tracing, and screening within the food chain [63]. However, NGS has a LoD of 5 × 10^4^ CFU/mL after 24 h enrichment, is expensive, requires trained personnel, and results are limited to the accuracy of the reference database used [63].

Matrix-Associated Laser Desorption Ionization-Time of Flight Mass Spectrometry (MALDI-TOF MS) is another promising technology that has been used for the identification and detection of *Salmonella* in food samples [64]. This method identifies microorganisms based on their unique protein profiles using a peptide mass fingerprint (PMF) database [65]. This allows for rapid identification and high accuracy but is culture-dependent and relies on the accuracy of reference databases [66]. The Bruker MALDI Biotyper method is a validated method for the detection of *Salmonella* in food samples that has been listed by the USDA Food Safety and Inspection Service (FSIS) [58,67]. Additionally, MALDI-TOF MS has also been used effectively in the poultry industry in Thailand as well as in an epidemiological study in Korea [68,69].

## 5. Emerging Technologies

Recent years have witnessed the emergence of novel detection technologies for *Salmonella* species in food, leveraging gene-editing, biosensors, and nanotechnology-based approaches [53]. Studies have combined conventional PCR amplification with a gene-editing system that utilizes clustered regularly interspaced short palindromic repeats (CRISPR) and associated proteins to enhance foodborne pathogen detection specificity and sensitivity [70]. In 2022, Wang and colleagues conducted one such study and suggested that their CRISPR/Cas9 integrated system could be used in on-site detection, as it provides accurate detection of *Salmonella* (log 2 CFU/mL) that can be visualized with the naked eye [70]. Furthermore, integrating NAA with technologies like CRISPR, microfluidic chips, biosensors, and nanotechnology may enhance the speed of detection as well as sensitivity and specificity [53,71,72].

### 5.1. Electrochemical Aptasensors

**Figure 1:**
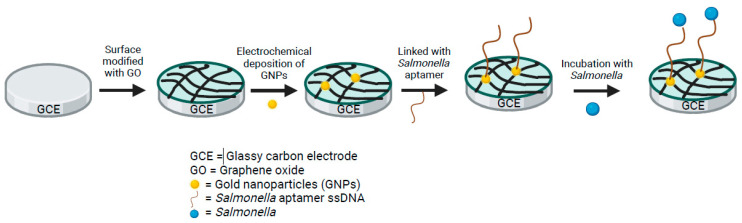
Schematic illustration of aptamer-based electrochemical biosensor construction used for detection of *Salmonella*. GCE was modified with GO and GNPs for biocompatibility and high electron transfer properties. Then, thiolated aptamer ssDNA was attached to the surface, capable of capturing *Salmonella*. Created in BioRender. Wolfram, A. (2024) https://BioRender.com/r18r866 (accessed on 23 November 2024).

Biosensor technology has gained popularity, as it can provide sensitive, reliable results and supply much higher speed with the additional benefit of bacteria inactivation directly on site [73]. Biosensors integrate biological recognition elements, such as antibodies or aptamers, with transducer platforms to enable real-time, label-free detection of *Salmonella* [74]. The specification of electrochemical biosensors determines the limit of detection (LoD) and detection time of the aptasensor platform. Aptasensors are frequently made with glassy carbon electrodes (GCE) and gold (Au) as the working electrode because of their high electrochemical stability, good biocompatibility with recognition molecules, and fast electron transfer for sensitive detection [73]. Adding nanomaterials onto the surface to make gold nanoparticles (AuNPs) enhances surface area, electron transfer kinetics, and electrical conductivity for more sensitive detection [73]. A 2022 study examined the performance of 15 published studies on electrochemical aptasensors for the detection of *Salmonella* species between 2014 and 2021 [74]. They found the developed sensors exhibited a LoD from 550 CFU/mL to as low as 1 CFU/mL (2 studies) within 5 to 240 min of detection time. Similarly, various sensor material combinations have been studied to enhance electron transfer properties and improve LoD and total detection time (Table 1). Aptasensors utilizing AuNPs decorated with graphite electrodes (GE) detected *Salmonella* in milk samples at 1 CFU/mL within 40 min [75] (Figure 1). In 2014, Ma and colleagues used GCE aptasensors modified with graphene oxide (GO) to detect *Salmonella* in pork samples with a LoD of 3 CFU/mL in 35 min [76]. In 2018, Ge and colleagues combined AuNPs with RCA to produce long DNA molecules and hybridize with the detection probes for enzyme-amplified readout on the surface of the aptasensor with a LoD of 16 CFU/mL in 1 h [77]. In 2019, Muniandy and colleagues utilized a reduced graphene oxide-titanium dioxide nanocomposite-based aptasensor to detect *S.* Typhimurium in just 5 min at a LoD of 10 CFU/mL [78]. Hasan and colleagues conducted a study in 2018 utilizing amino-modified ssDNA immobilized on multi-walled carbon nanotubes and found a LoD of 55 CFU/mL (for *S.* Enteritidis) and 67 CFU/mL (*S.* Typhimurium) in just 20 min. Taken together, electrochemical aptasensors’ portability, sensitivity, and rapid detection highlight their potential for widespread use for *Salmonella* detection in food safety and disease prevention. Future studies are needed on electrochemical aptasensor detection of other serovars of *Salmonella* like Typhi and Paratyphi to establish a quick, sensitive, cost-effective, and reliable technique for all *Salmonella* serovar detection [32,75,76,77,78,79].

### 5.2. Surface Plasmon Resonance

**Figure 2:**
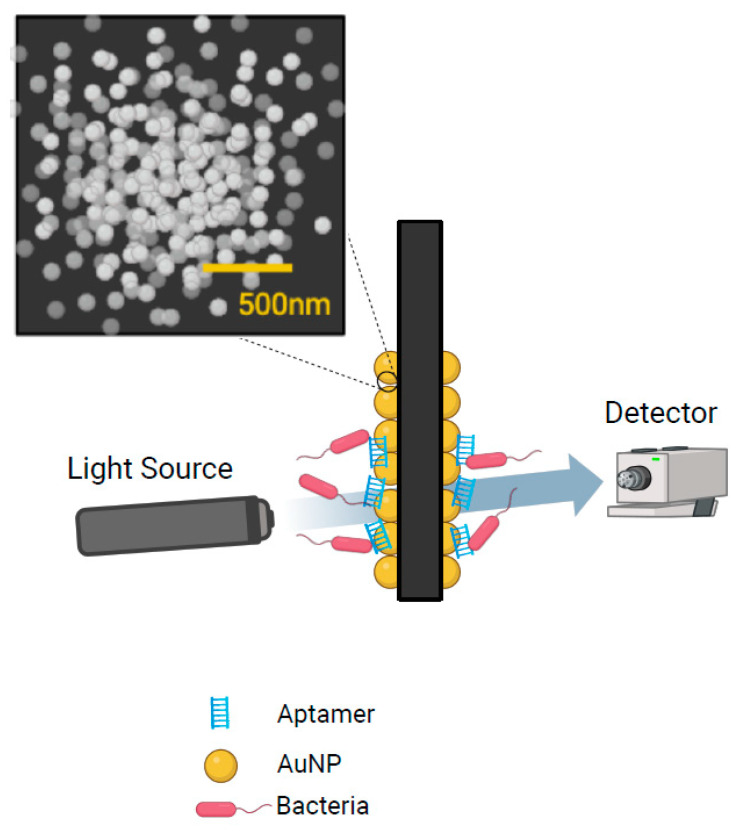
Schematic representation of the detection of bacteria using the gold nanoparticle-aptamer-based localized surface plasmon resonance (SPR) sensing chip. Created in BioRender. Wolfram, A. (2024) https://BioRender.com/c38m504 (accessed on 5 December 2024).

Another promising emerging technology that has gained a significant amount of interest is Surface Plasmon Resonance (SPR) (Table 2). SPR uses polarized light directed at a metal film to create surface plasmons that cause specific energy transfers to cause a reduction in light intensity called the SPR angle [80]. When biomolecules bind to the sensor surface, the SPR angle changes proportional to the mass of the bound molecules to provide real-time, label-free detection with high sensitivity and specificity without the need for sample enrichment [81]. In 2019, Bhandari and colleagues evaluated direct SPR assay, sequential two-step sandwich assay, and pre-incubation one-step sandwich assays for the detection of *Salmonella* Typhimurium in samples of romaine lettuce at different levels. They found that all three formats had a LoD of 5.9 log CFU/g without enrichment in just 2 min and 0.9 log CFU/g with a 24 h enrichment step [80]. In 2022, Bhandari and colleagues studied the SPR method with magnetic nanoparticles to detect *Salmonella* Typhimurium in romaine lettuce [81]. The authors demonstrated that the preincubation one-step sandwich assay had the highest signal amplification by forming complexes with antibody-coupled magnetic nanoparticles to significantly increase analyte mass. This increased sensitivity allowed a LoD for *S.* Typhimurium of 4.7 log CFU/mL in buffer and 5.2 log CFU/mL in romaine lettuce samples with the overall detection process from preparation to SPR detection completed in 4 h [81]. Oh and colleagues developed a gold nanoparticle-aptamer-based localized SPR sensing chip for the detection of *S.* Typhimurium in pork meat with a LoD of 4 log CFU/mL in 35 min and found no effect of food matrix or background contaminant microflora [82] (Figure 2). In 2024, Liang and colleagues were able to reduce the LoD of SPR to 42 CFU/mL by utilizing a sandwich strategy with antibody-AuNPs with a total detection and separation time of less than 50 min. [83]. The rapid detection time and no food matrix effect on detection are advantageous aspects of SPR, however, sensitivity of 4–5 log CFU/mL is insufficient in future food detection as methods with a sensitivity of 1 CFU/mL have been demonstrated [46,53,81,82,83].

### 5.3. Surface Enhanced Raman Spectroscopy

**Figure 3:**
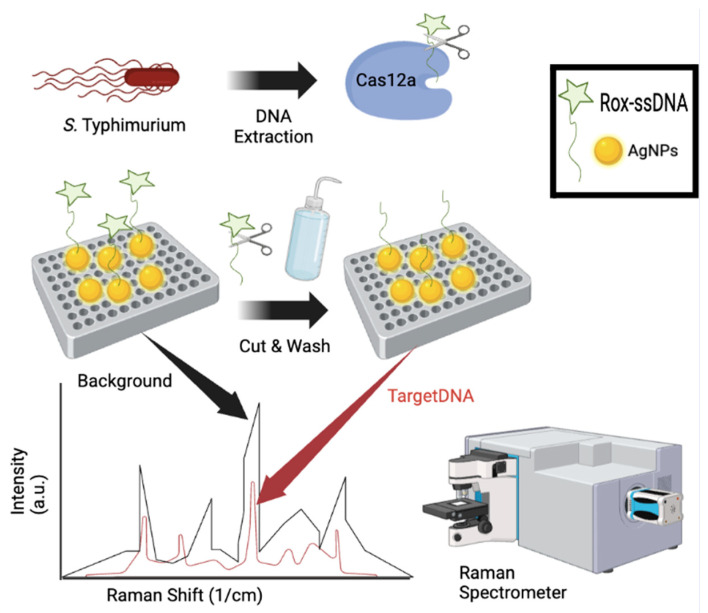
Schematic illustration of the CRISPR-SERS biosensor. DNA was extracted from *S.* Typhimurium and used to trigger the CRISPR system after binding with Cas12a-crRNA duplex for cleavage. The Raman signal reporter consists of ssDNA and Rox molecular which will be cleaved to decrease Raman intensity following wash out from SERS substrate. Without *S.* Typhimurium present, Cas12a/ccRNA would not initiate the cleavage activity of the probe, resulting in no detectable change in the Raman signal. Created in BioRender. Wolfram, A. (2024) https://BioRender.com/k99l567 (accessed on 23 November 2024).

Raman spectroscopy is a third example of an upcoming *Salmonella* species detection technology that functions by monitoring the changes in vibrations within functional groups in target molecules [84]. Similar to electrochemical biosensors, Raman spectroscopy has recently been enhanced via noble metal and nanotechnology to create Surface-Enhanced Raman Spectroscopy (SERS) (Table 3). SERS offers enhanced sensitivity and multiplexing capabilities for *Salmonella* detection in complex food matrices [84,85,86]. In 2022, Asgari and colleagues utilized a SERS optofulidic sensor coupled with immunoassay to selectively separate and detect a wide range of *E. coli* and *Salmonella* species in lettuce and packed salad samples. After only a 15 min enrichment period, their proposed protocol displayed promising results with a LoD of 10 CFU/200 g sample with total analysis time of just 2 h [84]. Chuesiang and colleagues conducted a study using aptamer-based SERS for detection of *Salmonella* in ground beef. However, their LoD (4 log CFU/g) and total running time (4 h) were not as promising as the previous study [85]. Recently, Wang and colleagues conducted a groundbreaking study using bi-channel lateral flow immunoassay strips with three-dimensional membrane SERS nanostickers (GO@Au/Ag) for multiplex detection of four bacteria in real clinical samples. Their proposed sensor displayed high accuracy, stability, and efficiency by quantitatively detecting four different bacteria with a LoD of 9 cells/mL in just 20 min [86]. However, future studies are needed on this sensor using food matrices for foodborne pathogen detection. In 2022, Zhuang and colleagues utilized a recombinase polymerase amplification (RPA)-integrated microfluidic paper-based analytical device (RPA-Cas12a-µPAD) for SERS-based detection of *S.* Typhimurium in contaminated meat and milk samples. The RPA-Cas12a-µPAD displayed accurate tests of food samples with a LoD of 3–4 CFU/mL in just 45 min [87]. In 2023, Jia and colleagues developed the CRISPR-SERS biosensor by combining CRISPR technology with SERS analytical method, aimed at detecting *invA* of *S.* Typhimurium. Results from their study showed that CRISPR-SERS could detect *S.* Typhimurium in poultry samples with a LoD of 110 CFU/mL within 2 h [88] (Figure 3). Likewise, Zheng and colleagues studied a novel SERS chip using aptamer-modified multi-color SERS tags and a graphene-coated plasmonic gold substrate to detect and inactivate *Salmonella* in whole blood samples [89]. Using Python to analyze their Raman mapping, they found that the SERS chip provided sensitive (4 CFU/mL), specific, and reproducible multiplex detection and in-situ photothermal elimination of pathogens (nearly 100% eradication reported), making it suitable for onsite multiplex pathogen detection and inactivation [89].

### 5.4. Bacteriophages

**Figure 4:**
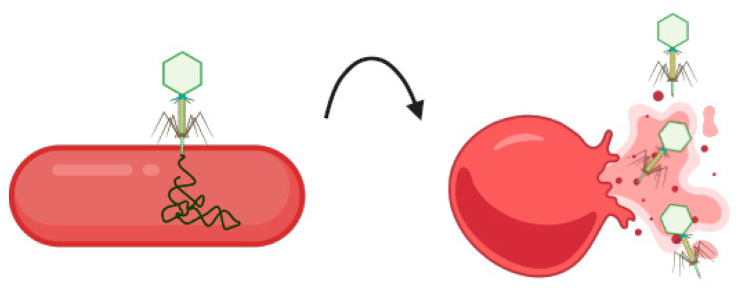
Schematic illustration of a bacteriophage detecting, infecting, and lysing bacteria like *Salmonella*. Created in BioRender. Wolfram, A. (2024) https://BioRender.com/g33l156 (accessed on 23 November 2024).

Another emerging technology that has been used in foodborne *Salmonella* detection is bacteriophage technology [90]. Bacteriophages are natural viruses that infect bacteria and are a viable alternative to current antimicrobials in the control of these pathogens as numerous studies have shown their ability to lyse multi-drug-resistant *Salmonella* isolates [90,91,92] (Figure 4). In 2023, Lopez-Garcia et al., tested the identification and lytic activity of 22 previously characterized *Salmonella* bacteriophages against a panel of 143 *Salmonella* isolates (Table 4) [93]. Phage STW-77 was the most effective overall, lysing 37.76% of all *Salmonella* isolates; SPFM5 and SPFM13 were most effective against *S.* Enteritidis, lysing 85.71% of isolates. STW-77 was most effective against *S.* Typhimurium and its monophasic variants. The effectiveness of phages varied within serovars, suggesting that phages may detect and lyse specific subtypes within a serovar. Unfortunately, no phage in this study could completely lyse the *S.* Kentucky ST198 MDR clones [93]. Although future studies are needed, these emerging technologies hold promise for future enhanced food safety monitoring and surveillance.

## 6. Challenges Associated with Detection Technologies: Limits of Detection and Enrichment Time

**Figure 5:**
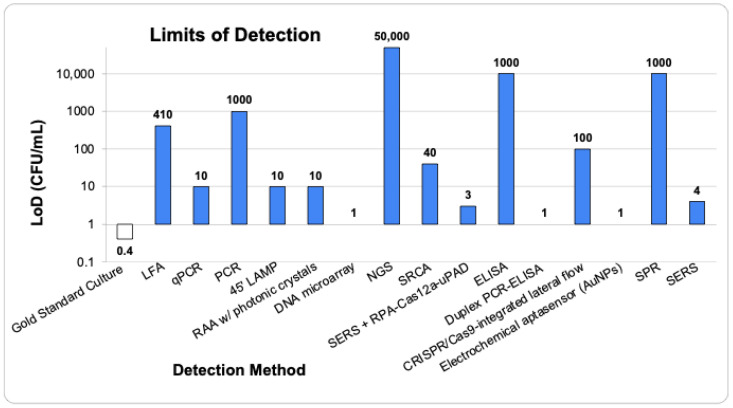
A comparison of the Limits of Detection (LoD) across various technologies used for the Detection of *Salmonella* species. All LoDs were converted to CFU/mL from CFU/g using 1g to 1 mL conversion based on the density of water.

**Figure 6:**
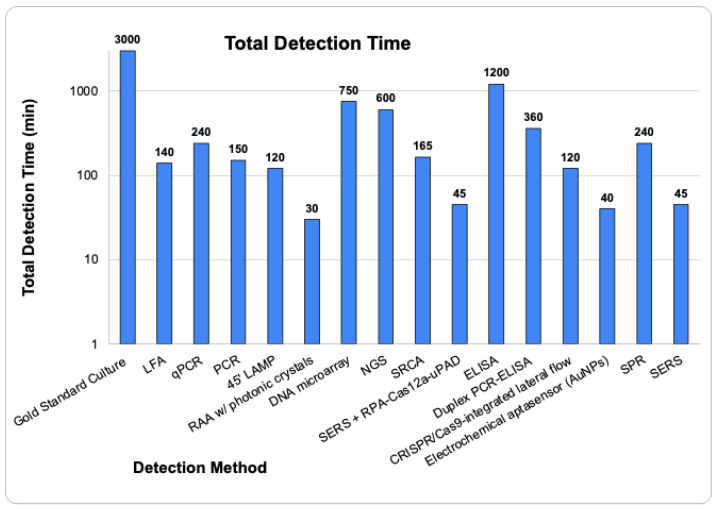
A comparison of the total time required across various technologies used for the detection of *Salmonella* species. The average reported detection time for each method (including any enrichment time) was used to create this chart.

**Figure 7:**
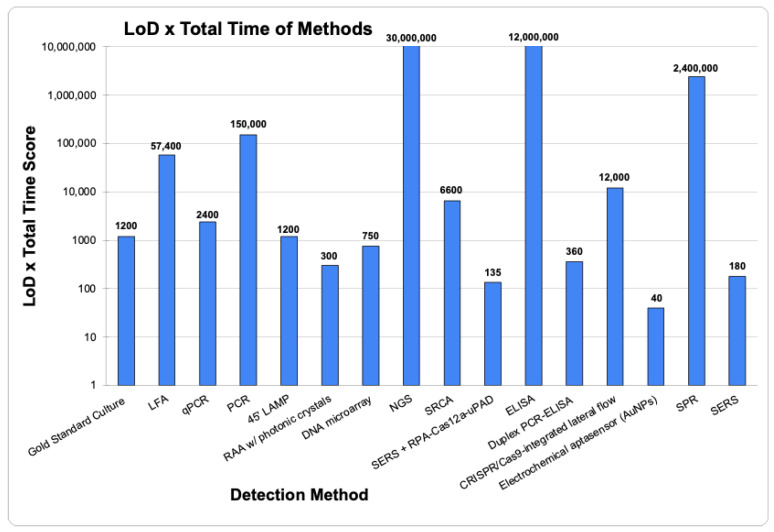
Factoring both Limits of Detection (LoD) and total detection time (including enrichment) for comparison of the various detection methods of *Salmonella* species. Lower total values have lower LoDs and total detection times. All LoDs were converted to CFU/mL from CFU/g using 1 g to 1 mL conversion based on the density of water.

According to the European Commission, the presence of *Salmonella* species at just one CFU/mL in a 25 g portion of ready-to-eat food is sufficient to cause disease in humans [1,9]. Emerging technologies such as LFA, PCR, NGS, ELISA, and SPR (Figure 5) have limits of detection (LoDs) significantly higher than the critical 1 CFU/mL level necessary to cause disease. While having a sensitive detection method is crucial, it is not the only factor to consider when selecting an on-site foodborne pathogen detection method (Figure 6). Traditional culture methods, though highly sensitive, are time-consuming [32,43,48,53,71,81,88]. Additionally, detection methods that require more than eight hours (480 min) are impractical for on-site use as they would necessitate multiple work shifts to complete, delaying the timely determination and control of pathogen contamination [48,81,88]. Thus, the ideal on-site foodborne pathogen detection method should be sensitive (detecting less than 10 CFU/mL), quick, cost-effective, and should not require complex equipment or highly trained personnel [70,81,88]. Furthermore, a comparison of detection methods by combining their LoD and total detection time to create a combined score (Figure 7) suggests that Electrochemical Aptasensors (AuNPs), SERS, and RAA with photonic crystals are among the top three emerging future technologies that may be employed for *Salmonella* species detection in food samples.

## 7. Future Directions

Significant advances have been made in food preservation methods that help eliminate food pathogens including storage at low temperatures, chemical preservatives, and vacuum packaging [48]. However, despite advances in food manufacturing and safety, regular outbreaks indicate that foodborne pathogens like *Salmonella* species remain a significant public health risk [1]. An exciting avenue has emerged with potential eradication techniques that can significantly change food safety measures. Using targeted approaches such as bacteriophage and photothermal effects, there is potential for eradicating *Salmonella* contamination from food supply chains [89,93]. The efficacy and practicality of these methods remain to be studied, but they appear promising. However, all proof-of-concept studies must be validated according to ISO 16140 standards and other requirements set out by regulatory agencies such as the FDA and EFSA. The aforementioned emerging technologies, including but not limited to, electrochemical aptasensors, SERS, and RAA, have the potential to significantly improve foodborne pathogen detection technology [48]. Additionally, further research on the implementation of these technologies at various stages of food preparation is required. By investing in rapid, sensitive, onsite emerging technologies early in the detection process, food companies can reduce the rate-limiting pretreatment step and prevent the need for other resource-intensive downstream confirmation tests [94]. Automated detection technologies would allow scalable detection to quickly screen large sample volumes, minimize labor costs, and potentially reduce the volume of goods that may need to be recalled or discarded later [95]. With the innovation of affordable and rapid testing methods, large-scale testing for *Salmonella* can be done for environmental monitoring as well as livestock and poultry testing.

## 8. Conclusions

Emerging technologies for *Salmonella* detection in food samples will ultimately enhance our ability to quickly and accurately identify contamination, thereby bolstering food safety and public health. Traditional culture methods, though the gold standard, are cost-effective but yet are time-consuming. In this review, we analyzed immunological methods, molecular methods, biosensors, and WGS on isolated strains to compare sensitivity and detection time. Of the technologies discussed, biosensors offer the highest sensitivity, capable of detecting as low as 1 CFU/mL within 40 min. Similarly, SERS can achieve similar sensitivity at 4 CFU/mL within 45 min [87], while RAA offers the quickest turnaround time of 30 min and has a LoD of 10 CFU/mL [54]. On the other hand, the Duplex PCR-ELISA method, although taking longer at 6 h, provides highly sensitive detection down to 1 CFU/mL, combining the specificity of PCR with the versatility of ELISA [39]. The aforementioned advanced methods combine speed, sensitivity, and accuracy, addressing critical requirements for effective on-site detection. Their integration into food safety protocols can significantly minimize health risks and economic impacts associated with *Salmonella* contamination. However, to implement these technologies for real-time in-field testing, the biggest challenge continues to be the need for highly trained staff and specialized equipment, which complicates transport and logistics. The integration of biosensors with cost-effective technologies such as LFAs can provide a cost-effective, highly sensitive, and rapid method of detection. Continued innovation, validation and adoption of these technologies will be pivotal in achieving practical, reliable, and comprehensive pathogen detection, ensuring safer food supply chains and better health outcomes against the threat of *Salmonella* contamination.

## Figures and Tables

**Table 1:** Recent studies using Electrochemical Aptasensors for *Salmonella* species detection.

Emerging Technologies: Electrochemical Aptasensors				
Sensor Material	Limit of Detection (CFU/mL)	Detection Time (Min)	Source	Author
Gold nanoparticle (AuNPs)/Graphite electrode (GE)	1	40	*S. enterica* in milk	[75]
Glassy Carbon Electrode (GCE)/Graphene oxide (GO)	3	35	*Salmonella* spp. in pork	[76]
Gold (Au)/AuNPs	16	60	*S.* Typhimurium in milk	[77]
Reduced Graphene oxide-titanium dioxide (rGO-TiO_2_)	10	5	*S.* Typhimurium in Chicken meat	[78]
Multi-walled carbon nanotubes (MWCNTs)	55 (*S.* Enteritidis); 67 (*S.* Typhimurium)	20	*S.* Enteritidis + Typhimurium in chicken meat	[79]

**Table 2:** Recent studies using Surface Plasmon Resonance with Magnetic Nanoparticles for *Salmonella* species Detection.

Emerging Technologies: Surface Plasmon Resonance (SPR)				
Sensor	Limit of Detection	Detection Time	Source	Author
SPR Biosensor	0.9 log CFU/g; 5.7 log CFU/g	24 h enrichment; <2 min detection	*S.* Typhimurium in romaine lettuce	[80]
Magnetic Nanoparticles Enhanced SPR	5.2 log CFU/g	4 h (3.9 h to prep, <2 min detection)	*S.* Typhimurium in romaine lettuce	[81]
Gold nanoparticle aptamer-based localized SPR	4 log CFU/mL	35 min	*S.* Typhimurium in pork meat	[82]
Sandwich Antibody-AuNP SPR	42 CFU/mL	<50 min	*S.* Typhimurium in milk	[83]

**Table 3:** Recent studies using Surface Enhanced Raman Spectroscopy for *Salmonella* species Detection.

Emerging Technologies: Surface Enhanced Raman Spectroscopy (SERS)				
Sensor	Limit of Detection	Total time of Detection	Source	Author
SERS optofluidic sensor coupled with immunoassay	10 CFU/200g	2 h (w/15 min enrichment)	*S. enterica* + *E. coli* strains in lettuce and packed salad samples	[84]
SERS + short-chain adenine and fluorescein molecule	4 log	4 h	*S.* Enteritidis and *S.* Gaminara in ground beef	[85]
GO@Au/Ag-based SERS-LFA	9 cells/mL	20 min	*S.* Typhimurium, *E*. *coli*, *S. aureus*, *L. monocytogenes* in human urine + blood	[86]
RPA-Cas12a-μPAD SERS	3–4 CFU/mL	45 min	*S.* Typhimurium in meat and milk	[87]
CRISPR-SERS	110 CFU/mL	2 h	*S.* Typhimurium in minced poultry	[88]
Background-free SERS w/sandwich configuration (+ 100% photothermal inactivation of all bacteria)	10 CFU/mL	90 min	*S.* Typhimurium & *S. aureus* in human blood	[89]

**Table 4:** Using Bacteriophages for *Salmonella* species Detection.

Emerging Technologies: Bacteriophages [93]			
Phage	Sensitivity for *S.* Enteritidis	Sensitivity for *S.* Typhimurium	Detection Time (Hours)
SPFM1	76.19%	33.33%	18
SPFM2	76.19%	40.00%	18
SPFM3	76.19%	26.67%	18
SPFM4	66.67%	33.33%	18
SPFM5	85.71%	33.33%	18
SPFM6	76.19%	33.33%	18
SPFM7	66.67%	33.33%	18
SPFM8	80.95%	33.33%	18
SPFM9	80.95%	20.00%	18
SPFM10	14.29%	No effect	18
SPFM11	66.67%	20.00%	18
SPFM12	14.29%	No effect	18
SPFM13	85.71%	40.00%	18
SPFM14	61.90%	26.67%	18
SPFM15	71.43%	26.67%	18
SPFM16	80.95%	26.67%	18
SPFM17	79.19%	26.67%	18
SPFM19	14.29%	No effect	18
SPFM20	71.43%	33.33%	18
SPFM21	71.43%	26.67%	18
STW-77	80.95%	66.67%	18
SEW-109	42.86%	40.00%	18
